# SARS-related Virus Predating SARS Outbreak, Hong Kong

**DOI:** 10.3201/eid1002.030533

**Published:** 2004-02

**Authors:** Bo Jian Zheng, Yi Guan, Ka Hing Wong, Jie Zhou, Kin Ling Wong, Betty Wan Y. Young, Li Wei Lu, Shui Shan Lee

**Affiliations:** * University of Hong Kong, Hong Kong Special Administration Region, People’s Republic of China; † Department of Health, Hong Kong Special Administrative Region, People’s Republic of China; ‡ Pamela Youde Nethersole Eastern Hospital, Hospital Authority, Hong Kong Special Administrative Region, People’s Republic of China

**Keywords:** SARS, SARS-related virus, previous exposure, serologic study, dispatch

## Abstract

Using immunofluorescence and neutralization assays, we detected antibodies to human severe acute respiratory syndrome–associated coronavirus (SARS-CoV) and/or animal SARS-CoV–like virus in 17 (1.8%) of 938 adults recruited in 2001. This finding suggests that a small proportion of healthy persons in Hong Kong had been exposed to SARS-related viruses at least 2 years before the recent SARS outbreak.

A novel coronavirus has been identified as the cause of the 2003 global outbreak of severe acute respiratory syndrome (SARS) (*1*–*5*). Genetic analysis and epidemiologic studies suggest that SARS coronavirus (CoV) was introduced into humans not long ago. Recently, SARS-CoV–like viruses were isolated in Himalayan palm civets and racoon dogs in a retail live animal market in Guangdong Province, southern China (*6*), and some of the animals tested had antibodies to SARS-CoV–like virus. Phylogenetic analysis showed that the SARS-CoV–like animal viruses were closely related to the viruses found in humans. Serologic surveillance demonstrated that, in the same market, approximately 40% of wild animal traders and 20% of animal slaughterers had antibodies to SARS-CoV or SARS-CoV–like animal virus, but none of them had had SARS-like symptoms in the past 6 months. These investigations raised questions about whether the presence of the animal SARS-CoV–like virus in the market was an isolated event or if this virus had been prevalent in the human population in southern China before the SARS outbreak. A retrospective serologic study was conducted to address these questions.

## The Study

Serum samples collected in May 2001 from 938 healthy Chinese adults in Hong Kong and 48 confirmed SARS patients diagnosed in February and March 2003 in Guangdong were studied. All serum samples were aliquoted and stored at −20°C. The healthy adults were totally asymptomatic persons randomly recruited after a telephone interview concerning hepatitis B virus. The signs and symptoms of the SARS patients met the World Health Organization’s definition for surveillance, and SARS-CoV infection had been confirmed virologically.

All serum samples were heated at 56°C for 30 minutes. Specific antibodies for SARS-CoV and SARS-CoV–like virus were tested by using immunofluorescence (IF) assay at 1:10 dilution on FRhK-4 cells infected with either a human SARS-CoV strain (GZ50) (*5*) or an animal SARS-CoV–like virus (SZ16) (*6*), as reported (*1*). For sera positive for anti–SARS-CoV or anti–SARS-CoV–like virus, the antibody titer was further determined by serial titration. The IF-positive serum samples were serially diluted from 1:20 to 1:640 and then mixed with 100 50% tissue culture infective dose (TCID)_50_ of the representative human or animal virus strains for a serum neutralization assay. After incubation for 1 hour at 37°C, the mixture was inoculated in triplicate onto 96-well plates of FRhK-4 cell cultures. The results were determined after 3-day incubation at 37°C.

Seventeen (1.8%) archived samples from healthy adults showed IF antibodies against the human virus, animal virus, or both (titer range 1:20 to 1:1,280) and were confirmed by serum neutralization assay. An additional six samples were IF-antibody positive at a 1:10 dilution to either animal or human viruses, but they were negative in neutralization assay and were treated as negative. The positive rate was highest in the group ages 51 to 60 years and appeared to be more prevalent in female (13/561, 2.3%) than male patients (4/377, 1.1%) (Table). Of the 17 seropositive serum samples, 10 were from housewives, retired, or unemployed persons; 6 were from clerks, unskilled workers, or students; and one was from a professional (Table). Most of the seropositive persons (13/17) had a higher IF or neutralization antibody titer to the animal virus than the human virus (Figure). By contrast, the control group, comprising convalescent-phase sera from 48 confirmed SARS patients recruited from hospitals in Guangdong, all showed positive antibody results for both human SARS-CoV and animal SARS-CoV–like viruses, but they invariably exhibited higher IF and neutralization antibody levels against the human virus than the animal virus (Figure).

**Table:** Distribution of age, gender, and occupation of SARS-CoV–seropositive adults recruited in 2001^a^

Age (y)	No. of positive/total (%)	No. of positive/total in males (%)	No. of positive/total in females (%)	Occupation groups^b^	No. of positive/total (%)		
17–30	2/162 (1.2)	0/73 (0)	2/89 (2.2)	1	10/367 (2.7)	
31–40	3/236 (1.3)	0/93 (0)	3/143 (2.1)	2	5/235 (2.1)	
41–50	6/283 (2.1)	1/100 (1.0)	5/183 (2.7)	3	2/221 (0.9)	
51–60	4/150 (2.7)	3/57 (5.3)	1/93 (1.1)	4	0/110 (0)	
>60	2/107 (1.9)	0/55 (0)	2/52 (3.8)	5	0/5 (0)	
Total	17/938 (1.8)	4/378 (1.1)	13/560 (2.3)		17/938 (1.8)	

^a^SARS-CoV, severe acute respiratory syndrome–associated coronavirus. ^b^Group 1: Housewives (235), retired persons (96), and unemployed persons (36); Group 2: clerks (141), students (40), and associate professionals (54); group 3: service workers (47), craft-related workers (41), machine operators (56), and unskilled workers (77); group 4: managers and administrators professionals (33), professionals (35), civil servants (9), and sales persons (33); group 5: undefined.

**Figure:**
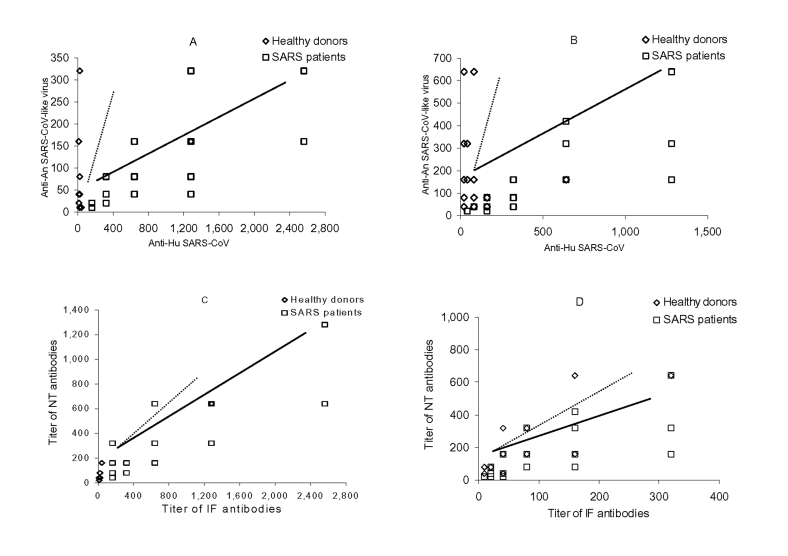
Correlation between antibodies against human severe acute respiratory syndrome cornonavirus (SARS-CoV) (anti-Hu SARS-CoV) and animal (anti-An) SARS-CoV–like virus in seropositive healthy adults recruited in 2001 (dotted line) and in patients with SARS in 2003 (thick line) by an immunofluorescence (A) and a neutralization (B) assay; and between neutralizing (NT) and immunofluorescence (IF) antibodies against Hu SARS-CoV (C) and a SARS-CoV–like virus (D).

## Conclusions

While the exposure history and symptoms of study participants were unavailable for assessment, our results suggest that a small portion of Hong Kong adults had acquired a SARS-CoV–related virus infection at least 2 years before the 2003 SARS outbreak. Cross-reactivity of the antibody to human SARS-CoV and the animal SARS-CoV–like virus must have occurred, in view of the marked similarity between the two viruses. Recently, we reported that the very similar sequences differed only by 60 to 80 nt, including an additional 29 nt in the animal virus (*6*). We speculate that the viruses that affected the 17 healthy persons >2 years ago were antigenically closer to the recently isolated animal SARS-CoV–like virus than human SARS-CoV, but interspecies transmission from animal to human was probably inefficient as the viruses might not have adapted in the new host. This hypothesis would explain why only a few persons became infected and why they were likely to be asymptomatic. Avian influenza is another example of a virus appearing first in animals before causing a human disease. While approximately 3%–10% of healthy persons who were in close contact with farm or market chicken or fowls showed positive antibody to avian influenza viruses at the time of the H5N1 outbreak in humans in 1997, none of them had symptoms of influenza (*7*).

Although human SARS-CoV and animal SARS-CoV–like viruses are related to the three families of coronaviruses that cause respiratory and gastrointestinal diseases in animals, phylogenetic analysis has shown that they are different enough to make up their own, fourth group. The number of members in this new group is not clear. Important factors in the emergence of novel infectious diseases from animal sources include extensive exposure and rapid virus evolution (*8*), which facilitate human-to-human transmission. The growth of the demand for wildlife in markets in Guangdong in the past 15 years has provided an ideal platform to facilitate interspecies virus transmission from animals to humans. Such factors could even directly trigger a zoonotic disease outbreak. Our observations distinguished two distinct serologic patterns. The high ratio of antibodies to the animal virus compared to the relatively low ratio of antibodies to the human virus in a small proportion of healthy adults >2 years ago signifies the circulation of a SARS-CoV–like virus and its ineffective propagation in the human population. Following rapid virus evolution and in the presence of an unknown trigger, the novel SARS-CoV may have effectively adapted to the human host, as illustrated by a second pattern characterized by a higher human-to-animal virus antibody titer in infected persons. Although this pilot study was limited by an unstandardized design of sample collection, our preliminary findings suggest that the occurrence of SARS might not be due to an isolated cross-species transmission event, but rather to the rapid evolution of a related virus that has taken root in the human population. This implies an expected pattern of potential SARS recurrence. Measuring the prevalence of the two antibodies in different species of animals and persons who had close contact with the animals is important to improve our understanding of SARS-CoV transmission dynamics.

## References

[1] Peiris JSM, Lai ST, Poon LL, Guan Y, Yam LY, Lim W, Coronavirus as a possible cause of severe acute respiratory syndrome. Lancet. 2003;361:1319–25. 10.1016/S0140-6736(03)13077-2 12711465 PMC7112372

[2] Poutanen SM, Low DE, Henry B, Finkelstein S, Rose D, Green K, Identification of severe acute respiratory syndrome in Canada. N Engl J Med. 2003;348:1995–2005. 10.1056/NEJMoa030634 12671061

[3] Ksiazek TG, Erdman D, Goldsmith C, Zaki SR, Peret T, Emery S, A novel coronavirus associated with severe acute respiratory syndrome. N Engl J Med. 2003;348:1953–66. 10.1056/NEJMoa030781 12690092

[4] Fouchier RA, Kuiken T, Schutten M, van Amerongen G, van Doornum GJ, van den Hoogen BG, Aetiology: Koch’s postulates fulfilled for SARS virus. Nature. 2003;423:240. 10.1038/423240a 12748632 PMC7095368

[5] Zhong NS, Zheng BJ, Li YM, Poon LLM, Xie ZH, Li PH, Epidemiological and aetiological studies of patients with severe acute respiratory syndrome (SARS) from Guangdong in February 2003. Lancet. 2003;362:1353–8. 10.1016/S0140-6736(03)14630-2 14585636 PMC7112415

[6] Guan Y, Zheng BJ, He YQ, Liu XL, Zhuang ZX, Cheung CL, Isolation and characterization of viruses related to the SARS coronavirus from animals in southern China. Science. 2003;302:276–8. 10.1126/science.1087139 12958366

[7] Bridges CB, Lim W, Hu-Primmer J, Sims L, Fukuda K, Mak KH, Risk of influenza A (H5N1) infection among poultry workers, Hong Kong, 1997–1998. J Infect Dis. 2002;185:1005–10. 10.1086/340044 11930308

[8] Holland JJ, de la Torre JC, Clarke DK, Duarte E. Quantitation of relative fitness and great adaptability of clonal populations of RNA viruses. J Virol. 1991;65:2960–7. 2033662 10.1128/jvi.65.6.2960-2967.1991 PMC240937

